# Wnt proteins as modulators of synaptic plasticity

**DOI:** 10.1016/j.conb.2018.06.003

**Published:** 2018-12

**Authors:** Faye McLeod, Patricia C Salinas

**Affiliations:** Department of Cell and Developmental Biology, University College London, Gower Street, London WC1E 6BT, UK

## Abstract

Dynamic changes in the structure and function of synapses in response to the environment, termed synaptic plasticity, are the cellular basis of learning and memory. At excitatory synapses, activation of NMDA receptors by glutamate leads to calcium influx triggering intracellular pathways that promote the trafficking of AMPA receptors to the post-synaptic membrane and actin remodeling. New evidence shows that Wnt secreted proteins, known for their role in synapse development, are essential for early stages of long-term potentiation, a form of plasticity that increases synaptic strength. Here, we review recent progress in this area and the significance of Wnt signaling to synaptic plasticity in health and disease.

## Highlights

• LTP induction promotes the localization of Wnt7a/b protein at dendritic spines.
• Wnt-Frizzled signaling is required for NMDA receptor-dependent LTP.
• Wnt7a specifically regulates rapid AMPA receptor trafficking at the synapse.
• Defects in Wnt signaling affect synaptic plasticity and integrity.

**Current Opinion in Neurobiology** 2018, **53**:90–95

This review comes from a themed issue on **Developmental neuroscience**

Edited by **Alex Kolodkin** and **Guillermina López-Bendito**

For a complete overview see the Issue and the Editorial

Available online 2nd July 2018

**https://doi.org/10.1016/j.conb.2018.06.003**

0959-4388/Crown Copyright © 2018 Published by Elsevier Ltd. This is an open access article under the CC BY license (http://creativecommons.org/licenses/by/4.0/).

## Introduction

Modulation in the strength of glutamatergic synapses in response to different inputs form the cellular basis of learning and memory [1]. Changes in the structure of dendritic spines, small protrusions that receive primarily excitatory input, and in the localization and function of ionotropic glutamate receptors (NMDA-type and AMPA-type) to spines are crucial for the modulation of synaptic efficacy [2, 3, 4]. Long-term potentiation (LTP) and long-term depression (LTD) are two established paradigms of synaptic plasticity, which are extensively studied in the hippocampus, an area of the brain essential for in learning and memory. LTP is defined as a long-lasting increase in synaptic strength whereas LTD is the opposite [1]. Both LTP and LTD last from minutes to days and research into the molecular events involved is essential for understanding the underlying mechanisms of memory formation.

**Figure 1:**
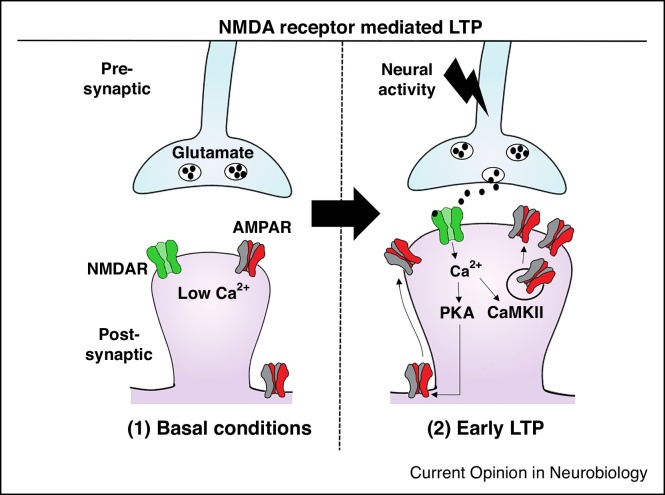
Key molecular mechanisms involved in NMDAR-mediated early LTP. (1) Under basal conditions, calcium (Ca^2+^) ion influx through NMDA receptors (NMDARs) is blocked by magnesium (Mg^2+^) ions in the pore. (2) An increase in neural activity following a specific stimulus pattern leads to enhanced glutamate release from the presynaptic terminal. Subsequently, glutamate binds to NMDARs on the post-synaptic side resulting in an influx of Ca^2+^ resulting in the activation of downstream signaling molecules including CaMKII and PKA promoting the exocytosis and lateral diffusion of AMPARs to the synapse. Spine size and synaptic strength are increased, which are essential for the expression of LTP.

Over the past 40 years, a vast amount of research has established the key molecular mechanisms involved in synaptic plasticity [5]. In this review, we will focus our attention solely on LTP. Although not exclusively, initiation of LTP is primarily NMDA receptor (NMDAR) dependent at the Shaffer collateral (SC)-CA1 cell synapse in the hippocampus [6]. Glutamate release from the pre-synaptic terminal binds to NMDARs and during repetitive synaptic activation and coincident postsynaptic depolarisation leads to relief of Mg^2+^ block of the NMDAR channel. Subsequently, an influx of Ca^2+^ and activation of Calmodulin-dependent protein kinase II (CaMKII) and Protein Kinase A (PKA) then occurs. Phosphorylation of AMPA receptors (AMPARs) and associated proteins by CaMKII results in an increase in the lateral diffusion and exocytosis of new AMPARs, increased dendritic spine size and elevated synaptic strength [7] (Figure 1). These molecular and structural changes are essential for the early stages of LTP (within 1 hour).

It is now widely accepted that LTP is directly influenced by the secreted factor brain-derived neutrophic factor (BDNF) [8^•^,^9^]. BDNF, which is regulated by neuronal activity, modulates synaptic AMPAR localization and promotes spine growth during LTP [8^•^,^9^]. The role of other synaptic modulators is less understood. For example, secreted proteins such as fibroblast growth factors contribute to LTP [10,11]. Moreover, recent studies demonstrate a role for Wnt proteins in synaptic plasticity, memory formation and synaptic integrity in the adult brain [12^••^,13^••^,14^••^]. In this review, we will discuss the emerging new roles for Wnts as key extracellular modulators of LTP.

## Wnt proteins in synapse formation

Wnts are a large family (19 members in humans and mice) of secreted glycolipoproteins that are evolutionarily conserved [15]. Historically, Wnts have been extensively studied for their critical role in embryonic patterning [16,17]. However, Wnts are also essential for axon pathfinding, dendritic development and the formation and function of synapses [16,18,19]. The function of Wnt signaling at the synapse was first established back in the late 1990s [20,21]. Subsequent studies cemented the contribution of Wnts to synapse development in different model systems [16,18,19,22,23]. Here, we will focus on their role at vertebrate central synapses.

Wnts promote pre-synaptic assembly. In the cerebellum, Wnt7a is expressed and released from granule cells to act retrogradely onto mossy fibre axons to regulate pre-synaptic assembly [20,21]. Supportive of these results, knockout mice deficient in Wnt7a and Dishevelled-1 (Dvl1), a scaffold protein essential for Wnt function [24], have defects in pre-synaptic differentiation at mossy fibre–granule cell synapses [25]. In hippocampal neurons, Wnt7a and its receptor Frizzled-5 (Fz5) are required for the formation of pre-synaptic sites [26]. Other Wnt proteins, such as Wnt5a, act through RAR-related orphan (RoR) receptors to increase the number of pre-synaptic sites on hippocampal neurons [27]. Furthermore, Wnt3a binds to Frizzled-1 (Fz1) receptors to regulate pre-synaptic protein clustering and vesicle recycling [28]. Collectively, these studies demonstrate a role for several Wnt proteins that signal through different receptors to promote pre-synaptic differentiation.

Wnts also signal to the dendrites to regulate post-synapse formation. *Wnt7a/Dvl1* deficient mice and gain of function studies *in vitro* using hippocampal neurons demonstrate that Wnt7a-Dvl1 signaling specifically promotes excitatory synapse formation and spine growth through CaMKII, without affecting inhibitory synapses [29]. Furthermore, these mice exhibit deficits in the frequency and amplitude of miniature excitatory postsynaptic currents (mEPSCs) [29]. As the amplitude of mEPSCs is a measure of the number of AMPARs in the postsynaptic membrane, these results suggest that Wnts not only act on the nerve terminal, but also signal postsynaptically to enhance synaptic strength. In contrast to Wnt7a, Wnt5a promotes inhibitory post-synaptic assembly by increasing GABA_A_ receptor clustering and enhancing the amplitude of inhibitory postsynaptic currents [29,30]. Although the *in vivo* role of Wnt5a at the synapse has not been reported, together these results suggest that members of the Wnt family differentially regulate excitatory and inhibitory post-synaptic properties. Moreover, Wnts act bidirectionally to promote the assembly of both sides of the synapse.

## Wnts in synaptic transmission

Electrophysiological recordings have demonstrated that Wnt proteins regulate synaptic transmission [25,29,31, 32, 33]. Pre-synaptically, *Wnt7a/Dvl1* mutant mice exhibit defects in neurotransmitter release at cerebellar mossy fibre–granule cell synapses [25] and at SC-CA1 synapses in the hippocampus [34]. Importantly, when the SC-CA1 synapse is activated by repetitive stimulation in *Wnt7a/Dvl1* mice, evoked transmitter release begins to fail within a few hundred milliseconds. In contrast, gain of function of Wnt7a promotes release in cultured neurons [32]. These findings demonstrate a role for Wnt7a signaling in neurotransmitter release. Post-synaptically, Wnt7a enhances synaptic strength by increasing the number of AMPA receptors at the post-synaptic membrane in hippocampal neurons [13^••^] Another Wnt protein, Wnt5a, potentiates postsynaptic NMDAR-mediated currents through RoR2 receptors in hippocampal neurons [31,33,35]. Thus, members of the Wnt family can modulate neurotransmission pre-synaptically and post-synaptically.

## Wnt signaling and synaptic plasticity

Activity-dependent structural and functional changes at the synapse are essential for the formation and refinement of neuronal networks during development and in the adult brain [36]. Several studies demonstrate that members of the Wnt family are regulated by neuronal activity [12^••^,13^••^,^26^,^31^,^37^, ^38^, ^39^, ^40^]. Studies at the *Drosophila* neuromuscular junction (NMJ) show that evoked activity induces the release of the Wnt1 homologue, Wingless [37]. Work using hippocampal neurons demonstrate that *Wnt2* mRNA is elevated following exposure to potassium chloride or bicuculline; general activity-enhancing stimulus paradigms [41]. Furthermore, Wnt3a protein levels are modulated by electrical stimulation in hippocampal slices [38] and NMDAR activation increases Wnt5a protein levels in cortical cultured neurons [40]. A recent study shows that Wnt7a/b protein levels are increased within 5 minutes at dendritic spines following tetanic stimulation in hippocampal acute slices and in cultured neurons [13^••^]. Consistent with this finding, the levels of Wnt7a/b protein are elevated in the adult hippocampus following environmental enrichment (EE) [39]. Activity also modulates the localization of Wnt receptors. For example, high frequency stimulation (HFS) leads to the increased localization of Fz5 receptors at the plasma membrane and at synapses [26]. Together, these studies demonstrate that neuronal activity modulates the mRNA and protein levels of Wnts and their receptors in different model systems.

Wnts and their receptors also play an important role in activity-dependent processes. Blockade of Wnts during HFS suppresses the recruitment of Fz5 to synapses [26]. Importantly, Wnt blockade completely abolishes activity-mediated synapse formation in cultured neurons [26]. Moreover, our recent study also demonstrates that blockade of endogenous Wnt proteins, with secreted frizzled related proteins (Sfrps), severely impairs LTP [13^••^]. Conversely, addition of Wnt proteins can facilitate LTP [12^••^,13^••^,^31^,^38^]. Together, these studies suggest that Wnts are key modulators of synaptic plasticity. New research has been focused on identifying the mechanisms by which Wnts modulate LTP.

Wnt5a has been shown to regulate NMDAR-mediated synaptic transmission in acute hippocampal slices [31]. However, the effect of this Wnt protein is slow, taking approximately 20 minutes to modulate NMDAR currents [31] whereas the initial potentiation of synaptic transmission during LTP happens within a few minutes. Although Wnt5a can influence the expression of LTP [31,42^•^], it does not affect endogenous synaptic AMPAR localization or dendritic spine size in hippocampal cultured neurons [13^••^]. Collectively, these findings suggest that Wnt5a may contribute to later stages of LTP.

A new study shows that Wnt7a/b regulate the early stages of NMDAR-dependent LTP [13^••^]. We found that acute blockade of endogenous Wnts with Sfrps reduces LTP induced by HFS in acute hippocampal slices or glycine-mediated chemical LTP (cLTP) in hippocampal neurons. Structural changes in dendritic spines, enhanced synaptic strength, and synaptic localization of AMPARs are all inhibited in the presence of Sfrps during LTP. Importantly, gain of function studies using single-particle tracking and super-ecliptic pHluorin-tagged AMPARs in hippocampal neurons demonstrate that Wnt7a rapidly (within 10 minutes) increases spine growth, synaptic AMPAR recruitment and synaptic strength, similar to the early stages of LTP [13^••^]. These results indicate that endogenous Wnt signaling is required for structural and functional plasticity during LTP.

**Figure 2:**
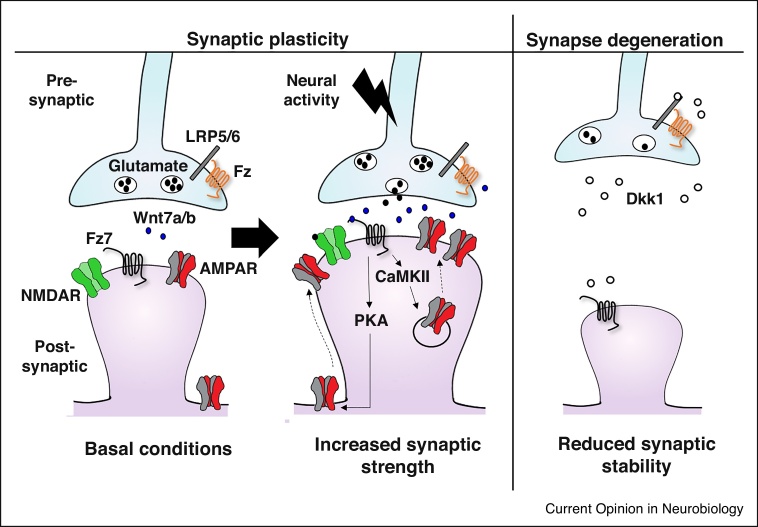
The role of Wnts in synaptic plasticity and synapse integrity. Synaptic plasticity: enhanced neural activity leads to increased levels of Wnt7a/b at CA1 synapses in the hippocampus. Pre-synaptic Wnt7a signaling enhances evoked transmitter release. Postsynaptically, Wnt7a/b binds to Fz7 receptors to activate CaMKII and PKA resulting in the increase of synaptic AMPAR localization, spine size and synaptic strength. Synapse degeneration: Wnt deficiency induced by expression of the Wnt antagonist Dkk1 or loss of function of LRP6 results in excitatory synapse degeneration, LTP deficits and long-term memory impairment in the adult hippocampus. The effect of Wnt deficiency on surface NMDA and AMPA receptor levels has not been determined. Dotted lines represent potential mechanisms. LRP5/6, Lipoprotein receptor-related protein 5/6.

What is the mechanism by which Wnt7a enhances synaptic AMPAR localization and strength? Wnt7a binds to Frizzled-7 (Fz7) and Fz5 receptors, which are both present at excitatory synapses in hippocampal neurons [13^••^,^26^]. In contrast to Fz5, Fz7 is detected on the post-synaptic side and regulates dendritic spine number under basal conditions [13^••^]. Loss of function studies in hippocampal neurons and acute slices demonstrate that Fz7 receptors are required for structural plasticity, AMPAR recruitment to spines and synaptic potentiation following LTP induction [13^••^]. Notably, Wnt7a requires Fz7 to regulate LTP induction. These studies also demonstrate that Wnt7a-Fz7 signaling promotes synaptic and extrasynaptic AMPAR localization through CaMKII and PKA activation, respectively [13^••^], which are central features of LTP. This work identifies Wnt7a-Fz7 signaling as a key pathway that regulates spine plasticity and synaptic accumulation of AMPARs during the early stages of LTP (Figure 2).

Recent studies using a mouse model that induces Wnt deficiency in the adult brain demonstrate that Wnt proteins are required for synapse stability and synaptic plasticity [12^••^,^43^]. Transgenic mice that inducibly express the specific Wnt antagonist Dickkopf-1 (Dkk1) secreted protein in the hippocampus exhibit loss of excitatory synapses, defects in LTP, enhanced LTD and deficits in long-term memory [12^••^] (Figure 2). Interestingly, acute blockade of endogenous Wnts by Sfrps does not affect excitatory synapse number but still inhibits LTP induction [13^••^,^34^]. This is not due to acute versus long-term exposure to Wnt antagonists, as Dkk1 also rapidly induces synapse loss in mature neurons [12^••^,^44^]. Although the mechanisms that lead to these differences are still unknown, both antagonists have a different mode of action. Sfrps inhibits Wnt function by binding to Wnt proteins [45] whereas Dkk1 blocks the Wnt co-receptor, lipoprotein receptor-related protein 6 (LRP6) [46] and therefore affects a specific Wnt signaling pathway (the canonical cascade). A proper balance between canonical and non-canonical Wnt signaling might determine whether synapses are lost or only their function is affected. Jointly, studies using these two Wnt antagonists have uncovered distinct functions for Wnts in synapse integrity and synaptic plasticity.

## Deficient Wnt signaling and synapse degeneration

Reduced synaptic plasticity and memory loss are key features of neurodegenerative conditions, including Alzheimer’s disease (AD). Synapse degeneration occurs early in AD and is highly correlated with cognitive deficits [47]. Several studies have demonstrated that oligomers of Amyloid-beta (Aß), the main component of Amyloid plaques, weaken synapses, decrease LTP and ultimately promotes synapse loss [48]. However, the molecular mechanisms by which Aß triggers these synaptic changes are poorly understood.

Mounting evidence suggests that impaired Wnt signaling contributes to AD pathology. First, expression of the endogenous Wnt antagonist Dkk1 is increased in the brain of AD patients [49]. Second, Aß rapidly induces the expression of Dkk1 and blockade of Dkk1 protects synapses against Aß [44]. Third, a variant of LRP6, a Wnt co-receptor that is blocked by Dkk1, has been linked to late onset AD [50] and deletion of LRP6 exacerbates pathology in an AD mouse model [14^••^]. These findings collectively with those obtained from mice that inducibly express Dkk1 [12^••^], demonstrate that deficient Wnt signaling affects synaptic integrity in the adult brain. In AD, decreased levels of Wnt signaling could weaken synaptic function resulting in the subsequent degeneration and loss of synapses characteristic of this condition.

An exciting and promising result is the finding that reactivation of Wnt signaling can restore connectivity after substantial synapse degeneration. Indeed, cessation of Dkk1 expression in transgenic mice that inducibly express Dkk1 fully restores the structural and functional plasticity and hippocampal dependent memory [12^••^]. In summary, these studies demonstrate the robust regenerative capacity of neurons in the adult hippocampus to assemble synapses within functional circuits after degeneration.

## Conclusion and outlook

This is an exciting time for the Wnt field in synaptic plasticity research. Although Wnts have been shown to play a crucial role in pre-synaptic and post-synaptic formation and synaptic transmission, new research has now identified Wnts as key modulators of the initial stages of LTP. Future work should focus on the mechanisms that control the release of Wnts during and following LTP induction and whether activity regulates release of Wnts in different brain areas and by different patterns of activity. For instance, Wnts could be stored and then released from exosomes at the synapse as observed at the *Drosophila* NMJ [51]. Whether similar mechanisms control Wnt release in vertebrates remain to be determined. Moreover, future studies might shed light into why different families of synaptic modulators have similar functions at the synapse. For example, Wnt and BDNF proteins could coordinate their activity or regulate each other during synaptic plasticity.

Deficient Wnt signaling could contribute to synaptic weakening, enhanced LTD and LTP deficits at early stages of AD. Boosting Wnt signaling could strengthen synapses resulting in their protection from toxic molecules such as Aß. Further research into the role of Wnts in synaptic plasticity will shed new light into potential mechanisms contributing to synaptic failure in neurological disorders.

## Conflict of interest statement

Nothing declared.

## Funding

This work was funded by the MRC (MR/M024083/1), Alzheimer’s Research UK (ARUK-PG2012-12), and the Wellcome Trust (089013/A/09/Z).

## References and recommended reading

Papers of particular interest, published within the period of review, have been highlighted as:

• of special interest
•• of outstanding interest

## References

[1] Takeuchi T., Duszkiewicz A.J., Morris R.G. (2014). The synaptic plasticity and memory hypothesis: encoding, storage and persistence. Philos Trans R Soc Lond B Biol Sci.

[2] Chater T.E., Goda Y. (2014). The role of AMPA receptors in postsynaptic mechanisms of synaptic plasticity. Front Cell Neurosci.

[3] Henley J.M., Wilkinson K.A. (2016). Synaptic AMPA receptor composition in development, plasticity and disease. Nat Rev Neurosci.

[4] Huganir R.L., Nicoll R.A. (2013). AMPARs and synaptic plasticity: the last 25 years. Neuron.

[5] Lomo T. (2018). Discovering long-term potentiation (LTP) — recollections and reflections on what came after. Acta Physiol (Oxf).

[6] Bliss T.V., Collingridge G.L. (2013). Expression of NMDA receptor-dependent LTP in the hippocampus: bridging the divide. Mol Brain.

[7] Herring B.E., Nicoll R.A. (2016). Long-term potentiation: from CaMKII to AMPA receptor trafficking. Annu Rev Physiol.

[8•] Harward S.C., Hedrick N.G., Hall C.E., Parra-Bueno P., Milner T.A., Pan E., Laviv T., Hempstead B.L., Yasuda R., McNamara J.O. (2016). Autocrine BDNF-TrkB signalling within a single dendritic spine. Nature.

[9] Leal G., Afonso P.M., Salazar I.L., Duarte C.B. (2015). Regulation of hippocampal synaptic plasticity by BDNF. Brain Res.

[10] Xiao M., Xu L., Laezza F., Yamada K., Feng S., Ornitz D.M. (2007). Impaired hippocampal synaptic transmission and plasticity in mice lacking fibroblast growth factor 14. Mol Cell Neurosci.

[11] Zhao M., Li D., Shimazu K., Zhou Y.X., Lu B., Deng C.X. (2007). Fibroblast growth factor receptor-1 is required for long-term potentiation, memory consolidation, and neurogenesis. Biol Psychiatry.

[12••] Marzo A., Galli S., Lopes D., McLeod F., Podpolny M., Segovia-Roldan M., Ciani L., Purro S., Cacucci F., Gibb A., Salinas P.C. (2016). Reversal of synapse degeneration by restoring Wnt signaling in the adult hippocampus. Curr Biol.

[13••] McLeod F., Bossio A., Marzo A., Ciani L., Sibilla S., Hannan S., Wilson G., Palomer E., Smart T.G., Gibb A., Salinas P.C. (2018). Wnt signaling mediates LTP-dependent spine plasticity and AMPAR localization through Frizzled-7 receptors. Cell Rep.

[14••] Liu C.C., Tsai C.W., Deak F., Rogers J., Penuliar M., Sung Y.M., Maher J.N., Fu Y., Li X., Xu H., Estus S., Hoe H.S., Fryer J.D., Kanekiyo T., Bu G. (2014). Deficiency in LRP6-mediated Wnt signaling contributes to synaptic abnormalities and amyloid pathology in Alzheimer’s disease. Neuron.

[15] Nusse R., Clevers H. (2017). Wnt/beta-catenin signaling, disease, and emerging therapeutic modalities. Cell.

[16] Budnik V., Salinas P.C. (2011). Wnt signaling during synaptic development and plasticity. Curr Opin Neurobiol.

[17] van Amerongen R., Nusse R. (2009). Towards an integrated view of Wnt signaling in development. Development.

[18] Dickins E.M., Salinas P.C. (2013). Wnts in action: from synapse formation to synaptic maintenance. Front Cell Neurosci.

[19] Oliva C.A., Montecinos-Oliva C., Inestrosa N.C. (2018). wnt signaling in the central nervous system: new insights in health and disease. Prog Mol Biol Transl Sci.

[20] Lucas F.R., Salinas P.C. (1997). WNT-7a induces axonal remodeling and increases synapsin I levels in cerebellar neurons. Dev Biol.

[21] Hall A.C., Lucas F.R., Salinas P.C. (2000). Axonal remodeling and synaptic differentiation in the cerebellum is regulated by WNT-7a signaling. Cell.

[22] Packard M., Koo E.S., Gorczyca M., Sharpe J., Cumberledge S., Budnik V. (2002). The *Drosophila* Wnt, wingless, provides an essential signal for pre- and postsynaptic differentiation. Cell.

[23] Speese S.D., Budnik V. (2007). Wnts: up-and-coming at the synapse. Trends Neurosci.

[24] Gao C., Chen Y.G. (2010). Dishevelled: the hub of Wnt signaling. Cell Signal.

[25] Ahmad-Annuar A., Ciani L., Simeonidis I., Herreros J., Fredj N.B., Rosso S.B., Hall A., Brickley S., Salinas P.C. (2006). Signaling across the synapse: a role for Wnt and Dishevelled in presynaptic assembly and neurotransmitter release. J Cell Biol.

[26] Sahores M., Gibb A., Salinas P.C. (2010). Frizzled-5, a receptor for the synaptic organizer Wnt7a, regulates activity-mediated synaptogenesis. Development.

[27] Paganoni S., Bernstein J., Ferreira A. (2010). Ror1-Ror2 complexes modulate synapse formation in hippocampal neurons. Neuroscience.

[28] Varela-Nallar L., Grabowski C.P., Alfaro I.E., Alvarez A.R., Inestrosa N.C. (2009). Role of the Wnt receptor Frizzled-1 in presynaptic differentiation and function. Neural Dev.

[29] Ciani L., Boyle K.A., Dickins E., Sahores M., Anane D., Lopes D.M., Gibb A.J., Salinas P.C. (2011). Wnt7a signaling promotes dendritic spine growth and synaptic strength through Ca(2)(+)/Calmodulin-dependent protein kinase II. Proc Natl Acad Sci U S A.

[30] Cuitino L., Godoy J.A., Farias G.G., Couve A., Bonansco C., Fuenzalida M., Inestrosa N.C. (2010). Wnt-5a modulates recycling of functional GABAA receptors on hippocampal neurons. J Neurosci.

[31] Cerpa W., Gambrill A., Inestrosa N.C., Barria A. (2011). Regulation of NMDA-receptor synaptic transmission by Wnt signaling. J Neurosci.

[32] Cerpa W., Godoy J.A., Alfaro I., Farias G.G., Metcalfe M.J., Fuentealba R., Bonansco C., Inestrosa N.C. (2008). Wnt-7a modulates the synaptic vesicle cycle and synaptic transmission in hippocampal neurons. J Biol Chem.

[33] McQuate A., Latorre-Esteves E., Barria A. (2017). A Wnt/calcium signaling cascade regulates neuronal excitability and trafficking of NMDARs. Cell Rep.

[34] Ciani L., Marzo A., Boyle K., Stamatakou E., Lopes D.M., Anane D., McLeod F., Rosso S.B., Gibb A., Salinas P.C. (2015). Wnt signalling tunes neurotransmitter release by directly targeting Synaptotagmin-1. Nat Commun.

[35] Cerpa W., Latorre-Esteves E., Barria A. (2015). RoR2 functions as a noncanonical Wnt receptor that regulates NMDAR-mediated synaptic transmission. Proc Natl Acad Sci U S A.

[36] Cohen S., Greenberg M.E. (2008). Communication between the synapse and the nucleus in neuronal development, plasticity, and disease. Annu Rev Cell Dev Biol.

[37] Ataman B., Ashley J., Gorczyca M., Ramachandran P., Fouquet W., Sigrist S.J., Budnik V. (2008). Rapid activity-dependent modifications in synaptic structure and function require bidirectional Wnt signaling. Neuron.

[38] Chen J., Park C.S., Tang S.J. (2006). Activity-dependent synaptic Wnt release regulates hippocampal long term potentiation. J Biol Chem.

[39] Gogolla N., Galimberti I., Deguchi Y., Caroni P. (2009). Wnt signaling mediates experience-related regulation of synapse numbers and mossy fiber connectivities in the adult hippocampus. Neuron.

[40] Li Y., Li B., Wan X., Zhang W., Zhong L., Tang S.J. (2012). NMDA receptor activation stimulates transcription-independent rapid wnt5a protein synthesis via the MAPK signaling pathway. Mol Brain.

[41] Wayman G.A., Impey S., Marks D., Saneyoshi T., Grant W.F., Derkach V., Soderling T.R. (2006). Activity-dependent dendritic arborization mediated by CaM-kinase I activation and enhanced CREB-dependent transcription of Wnt-2. Neuron.

[42•] Chen C.M., Orefice L.L., Chiu S.L., LeGates T.A., Hattar S., Huganir R.L., Zhao H., Xu B., Kuruvilla R. (2017). Wnt5a is essential for hippocampal dendritic maintenance and spatial learning and memory in adult mice. Proc Natl Acad Sci U S A.

[43] Galli S., Lopes D.M., Ammari R., Kopra J., Millar S.E., Gibb A., Salinas P.C. (2014). Deficient Wnt signalling triggers striatal synaptic degeneration and impaired motor behaviour in adult mice. Nat Commun.

[44] Purro S.A., Dickins E.M., Salinas P.C. (2012). The secreted Wnt antagonist Dickkopf-1 is required for amyloid beta-mediated synaptic loss. J Neurosci.

[45] Kawano Y., Kypta R. (2003). Secreted antagonists of the Wnt signalling pathway. J Cell Sci.

[46] Niehrs C. (2006). Function and biological roles of the Dickkopf family of Wnt modulators. Oncogene.

[47] Arendt T. (2009). Synaptic degeneration in Alzheimer’s disease. Acta Neuropathol.

[48] Mucke L., Selkoe D.J. (2012). Neurotoxicity of amyloid beta-protein: synaptic and network dysfunction. Cold Spring Harb Perspect Med.

[49] Caricasole A., Copani A., Caraci F., Aronica E., Rozemuller A.J., Caruso A., Storto M., Gaviraghi G., Terstappen G.C., Nicoletti F. (2004). Induction of Dickkopf-1, a negative modulator of the Wnt pathway, is associated with neuronal degeneration in Alzheimer’s brain. J Neurosci.

[50] De Ferrari G.V., Papassotiropoulos A., Biechele T., Wavrant De-Vrieze F., Avila M.E., Major M.B., Myers A., Saez K., Henriquez J.P., Zhao A., Wollmer M.A., Nitsch R.M., Hock C., Morris C.M., Hardy J., Moon R.T. (2007). Common genetic variation within the low-density lipoprotein receptor-related protein 6 and late-onset Alzheimer’s disease. Proc Natl Acad Sci U S A.

[51] Budnik V., Ruiz-Canada C., Wendler F. (2016). Extracellular vesicles round off communication in the nervous system. Nat Rev Neurosci.

